# Searching for novel biomarkers using a mouse model of *CLN3*-Batten disease

**DOI:** 10.1371/journal.pone.0201470

**Published:** 2018-08-07

**Authors:** Derek Timm, Jacob T. Cain, Ryan D. Geraets, Katherine A. White, Seung yon Koh, Tammy Kielian, David A. Pearce, Michelle L. Hastings, Jill M. Weimer

**Affiliations:** 1 Pediatrics and Rare Diseases Group, Sanford Research, Sioux Falls, South Dakota, United States of America; 2 The University of South Dakota, Sioux Falls, South Dakota, United States of America; 3 Department of Pathology and Microbiology, University of Nebraska Medical Center, Omaha, Nebraska, United States of America; 4 Center for Genetic Disease, Department of Cell Biology and Anatomy, Chicago Medical School, Rosalind Franklin University of Medicine and Science, North Chicago, Illinois, United States of America; King’s College London, UNITED KINGDOM

## Abstract

*CLN3*-Batten disease is a rare, autosomal recessive disorder involving seizures, visual, motor and cognitive decline, and premature death. The *Cln3*^*Δex7/8*^ mouse model recapitulates several phenotypic characteristics of the most common 1.02kb disease-associated deletion. Identification of reproducible biomarker(s) to facilitate longitudinal monitoring of disease progression and provide readouts for therapeutic response has remained elusive. One factor that has complicated the identification of suitable biomarkers in this mouse model has been that variations in animal husbandry appear to significantly influence readouts. In the current study, we cross-compared a number of biological parameters in blood from *Cln3*^*Δex7/8*^ mice and control, non-disease mice on the same genetic background from multiple animal facilities in an attempt to better define a surrogate marker of *CLN3*-Batten disease. Interestingly, we found that significant differences between Batten and non-disease mice found at one site were generally not maintained across different facilities. Our results suggest that colony variation in the *Cln3*^*Δex7/8*^ mouse model of *CLN3*-Batten disease can influence potential biomarkers of the disease.

## Introduction

Batten disease or Neuronal Ceroid Lipofuscinoses (NCLs) is a family of lysosomal storage disorders sharing the common features of neurodegeneration and premature death, which are the result of mutations in as many as 14 different genes [1–3]. The most common childhood forms of Batten disease result from mutations in either *CLN1* (Classic Infantile Neuronal Ceroid Lipofuscinosis; cINCL; Haltia–Santavuori), *CLN2* (Classic Late-Infantile Neuronal Ceroid Lipofuscinosis; cLINCL; Janský–Bielschowsky) or *CLN3* (Classic Juvenile Neuronal Ceroid Lipofuscinosis; cJNCL; Spielmeyer–Sjögren). Disease onset for these variants ranges from infancy to adulthood depending on the affected gene and particular mutation [1, 2]. In this study, we focus on *CLN3*-Batten disease, which has symptomatic onset between 5–10 years of age; it most often begins with progressive blindness and intractable seizures, followed by cognitive and motor deterioration, and ultimately premature death by the 20s-early 30s [1]. Although significant advances in the treatment of other lysosomal storage disorders have been made over the last decade, therapeutics for *CLN3*-Batten disease do not currently exist [4]. However, over the last several years a number of clinical trials have been initiated for some of the NCLs (NCT01399047, NCT01161576, NCT01414985, NCT01907087, NCT00151216, NCT00337636, NCT00028262, NCT01238315, NCT02725580; ClinicalTrials.gov; reviewed in [4]) and, more recently, the FDA approved the first treatment for *CLN2*-Batten disease, an enzyme replacement therapy called Brineura[5].

Given the limited number of Batten disease patients [an estimated one in 12,500 live births in Anglo-Saxon countries and one in 100,000 worldwide (reviewed in [2, 6, 7]], there are a number of unique challenges facing researchers in the successful design of clinical trials studying such rare diseases. One advantage within the Batten disease community is access to a well-documented natural history of the various forms of the disease [1]. Additionally, comprehensive clinical rating scales have been developed that allow a physician to track patient progression both pre- and post-treatment [8, 9]. Furthermore, with advances in neurological imaging, clinicians have formulated metrics for monitoring MRI, CT or PET scans in patients enrolled in clinical trials. Unfortunately, these techniques often require sedation, which is extremely stressful for pediatric patients; further confounding the outcomes of clinical trials. Thus, the Batten disease field is still searching for reliable, non-invasive, biological parameters for tracking disease progression. Surrogate markers of disease (i.e., biomarkers) are useful tools that allow clinicians to collect patient tissue samples (i.e., blood or urine, buccal swabs, and skin biopsies) with minimal to no sedation, and to monitor changes over time. Identification of easily assessable disease markers is needed for the advancement of Batten disease therapeutic discovery. Preliminary feasibility of this approach has been previously demonstrated in hematological proteins in human Batten disease patient blood samples [10].

To expedite the identification of useful surrogate markers, we and others have turned to a mouse model of *CLN3*-Batten disease. CLN3 is a transmembrane lysosomal/late endosomal protein of unknown function, although its cellular localization suggests a putative role in endocytosis, autophagy, lysosomal transport, and/or apoptosis [11]. The *Cln3*^*Δex7/8*^ mouse model encompasses the most common *CLN3* mutation, namely a 1.02 kb deletion spanning exons 7 and 8 of *CLN3*[12–14]. This mouse model also recapitulates many phenotypic similarities with human *CLN3*-Batten disease, including accumulation of autofluorescent storage material, neuronal degeneration, glial activation, and behavioral deficits[12, 15–17]. In an attempt to identify surrogate markers aimed at tracking *CLN3*-Batten disease progression, Staropoli et al. utilized a comprehensive panel of biochemical assays to analyze peripheral tissues of *Cln3*^*Δex7/8*^ mice [18]. They reported changes in serum ferritin concentrations, red blood cell (RBC) mean corpuscular volume (MCV), and reticulocyte counts as well as decreased T cell numbers (the latter in male mice only) in *Cln3*^*Δex7/8*^ compared to wild-type mice [18].

While the search for relevant biomarkers has been encouraging, it has become increasingly evident over the past several years that disease markers in animal models, including *Cln3*^*Δex7/8*^ mice, can be affected not only by age, gender, and genetic background of the test animals, but also by seemingly innocuous differences in environmental conditions [19, 20]. Thus, for *CLN3*-Batten disease surrogate biomarkers to have the broadest utility in clinical applications, they must correlate with the disease regardless of the confounding effect of differing external factors. In this study, we explored whether the potential biomarkers of *CLN3*-Batten that have been previously identified remain consistent across *Cln3*^*Δex7/8*^ mice raised and housed at different research institutions (i.e., colonies in South Dakota, Nebraska, and Illinois), and also tested for new biomarkers that may be robust in predicting disease [18].

## Materials and methods

### Animal use

Animal protocols were approved by the Institutional Animal Care and Use Committees of each participating institute [NIH/OLAW Assurance Number: A4568-01 (Sanford); A3279-01 (Rosalind Franklin); A3294-01 University of Nebraska Medical Center)], with all procedures conducted in strict accordance with National Institutes of Health guidelines and Institutional Animal Care and Use Committee Guidelines at each of the three institutions. Wild-type and homozygous *Cln3*^Δex7/8^ mice, developed by Cotman et al. [12] on a C57BL/6J background, were initially obtained from Jackson Laboratory by Sanford Research and animals from this parental colony were then distributed to laboratories at Rosalind Franklin University of Medicine and Science and The University of Nebraska Medical Center. The mice used at Rosalind Franklin University of Medicine and Science were two to three generations from the Sanford/Jackson Laboratory founders, and the mice used at The University of Nebraska Medical Center were five to eight generations from the Sanford/Jackson Laboratory founders. Given male Batten disease patients often present with an earlier disease phenotype, and the Batten disease field has historically studied primarily male mice, for the studies presented here only male mice were used [1, 21, 22]. Animals housed at Sanford Research utilized irradiated Teklad 2018 chow (ad libitum), non-acidified water (ad libitum), Teklad 7092–7097 corncob bedding, and Teklad 7099 laboratory animal bedding as enrichment (Teklad, East Millstone, NJ). Mice were exposed to a 12-hour light/dark cycle. Animals from Rosalind Franklin University were provided with irradiated Envigo Teklad 2019S Global 19% protein extruded rodent diet, water filtered by reverse osmosis and delivered using a central Edstrom automated watering system (ad libitum), and Harlan 7090 Sani Chips-Aspen bedding, as well as Ancare cotton Nestlets (NES3600) and mouse walk up huts (Otto Environmental, JW-82100) for enrichment and exposed to a 12-hour light/dark cycle. Animals from the University of Nebraska Medical Center were provided with autoclaved Teklad Diet 2019 (ad libitum) and non-acidified water packaged using a Hydropac^™^ Watering System (Lab Products, Inc., Seaford, DE), and Bed ‘O cobs ¼ inch (The Andersons Inc., Maumee, OH) autoclaved bedding.

### Complete blood counts

A total of 61 mice from two of the colonies (32 from the University of Nebraska Medical Center, and 29 from Sanford Research) were analyzed for complete blood counts (CBC’s) at 1, 3, 6, and 12 months-of-age. Submandibular blood collection was performed as previously described [23]. Briefly, mice were restrained, and a lancet was used to puncture the submandibular vein for blood collection into EDTA-coated microtainer tubes (BD, Franklin Lakes, NJ). All samples were analyzed on the day of collection. CBC’s were performed using a Scil Vet ABC Hematology Analyzer^®^ following the manufacturer’s suggested protocol with the mouse species card (Scil Vet, Gurnee, IL).

### Clinical blood chemistry profiling

A total of 28 mice from three different colonies (Four each of mutant, homozygous *Cln3*^Δex7/8^ and wild-type from the University of Nebraska Medical Center, 10 from Rosalind Franklin University, and five each of mutant and wild-type from Sanford Research), all 5 months of age, were sent to Charles River Laboratories for clinical chemistry testing. Serum was analyzed for the following metabolites and biomarkers: Alanine aminotransferase, Albumin, Alkaline phosphatase, Aspartate aminotransferase, Bilirubin (total), Blood Urea Nitrogen, Calcium, Chloride, Cholesterol (Total), Creatinine, Ferritin, Gamma Glutamyl Transferase, Glucose, Inorganic Phosphorus (Phosphate), Iron, Potassium, Sodium, Total Iron Binding Capacity, Total Protein, Transferrin, and Triglycerides. These tests are included in the complete and iron clinical chemistry panels at Charles River Laboratories (http://www.criver.com/products-services/safety-assessment/pathology/clinical-pathology).

### Statistics

Statistics were performed using GraphPad Prism 6.04 and R 3.5.0. Specific statistical tests used are indicated in the figure legends. In general, either an unpaired, two-tailed t-test,ordinary two-way ANOVA, or ordinary three-way ANOVA was employed (reporting type III sum of squares). Statistical outliers were removed with the ROUT method, Q = 1%. For the unpaired, two-tailed t-test, variance was determined using the *F*-test. For the ordinary two-way ANOVA, variance was determined using the Brown-Forsythe test and post-hoc analysis was conducted within each individual blood parameter using a Tukey correction in Graph Pad Prism. The three-way ANOVA was performed in R 3.5.0 and was followed by Tukey’s Honest Significant Differences post hoc test. All data and ANOVA tables are available in S1, S2 and S3 Files. All graphs are presented as mean +/- 95% confidence interval, with mice from Sanford Research denoted as Colony 1, mice from The University of Nebraska Medical Center denoted as Colony 2, and mice from Rosalind Franklin University of Medicine and Science denoted as Colony 3. *p<0.05, **p<0.01, ***p<0.001, ****p<0.0001.

## Results

### Basic iron panel

**Fig 1:**
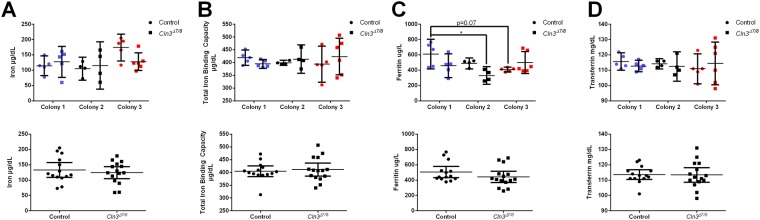
Significant interaction of colony and genotype on ferritin levels between three colonies at 5 months of age. The basic iron panel values of blood collected from each genotype was analyzed for colonies 1 (blue, Sanford Research), 2 (black, University of Nebraska Medical Center), and 3 (red, Rosalind Franklin University of Medicine and Science) for both control (circle) and *Cln3*^*Δex7/8*^ mice (square). Graphs indicate concentration of iron, (A), total iron binding capacity (B), ferritin (C), and transferrin (D), with individual colony data on top and pooled colony data on the bottom. A significant main effect of colony was detected on iron levels, p = 0.0388. (A) A significant interaction between colony and genotype was detected on ferritin levels, p = 0.0249; the colony factor approached significance at p = 0.0677 and the genotype factor had a p-value of 0.0922 (C). Data represented as mean ± 95% CI; data points represent individual mice from indicated colony. Statistical significance for top graphs was determined using ordinary two-way ANOVA, followed by Tukey’s multiple comparison test with a Bonferroni correction. Statistical significance for bottom, pooled colony graphs was determined using an unpaired student’s t test. *p<0.05.

Basic iron panels were used to assess hematological differences in iron, ferritin, transferrin, and total iron binding capacity. To assess the influence of external factors, the data was separated based on the home institution of the mouse colonies. This assessment indicated no significant differences in iron, ferritin, transferrin, or total iron binding capacity between each colony of wild-type and *Cln3*^*Δex7/8*^ mice. However, there was a significant interaction between Genotype and Colony on ferritin levels (p = 0.0249) and a significant main effect of Colony on iron levels (p = 0.0388) (Fig 1A–1D). When data from all three colonies were pooled, there were no significant differences detected in any of the parameters measured (Fig 1A–1D, bottom). There were no significant changes seen in basic iron panels when comparing control and *Cln3*^*Δex7/8*^ mice within a single colony, the only significant difference between control and *Cln3*^*Δex7/8*^ mice was between the control mice of colony 1 and the *Cln3*^*Δex7/8*^ mice of colony 2.

### Basic lipid panel

**Fig 2:**
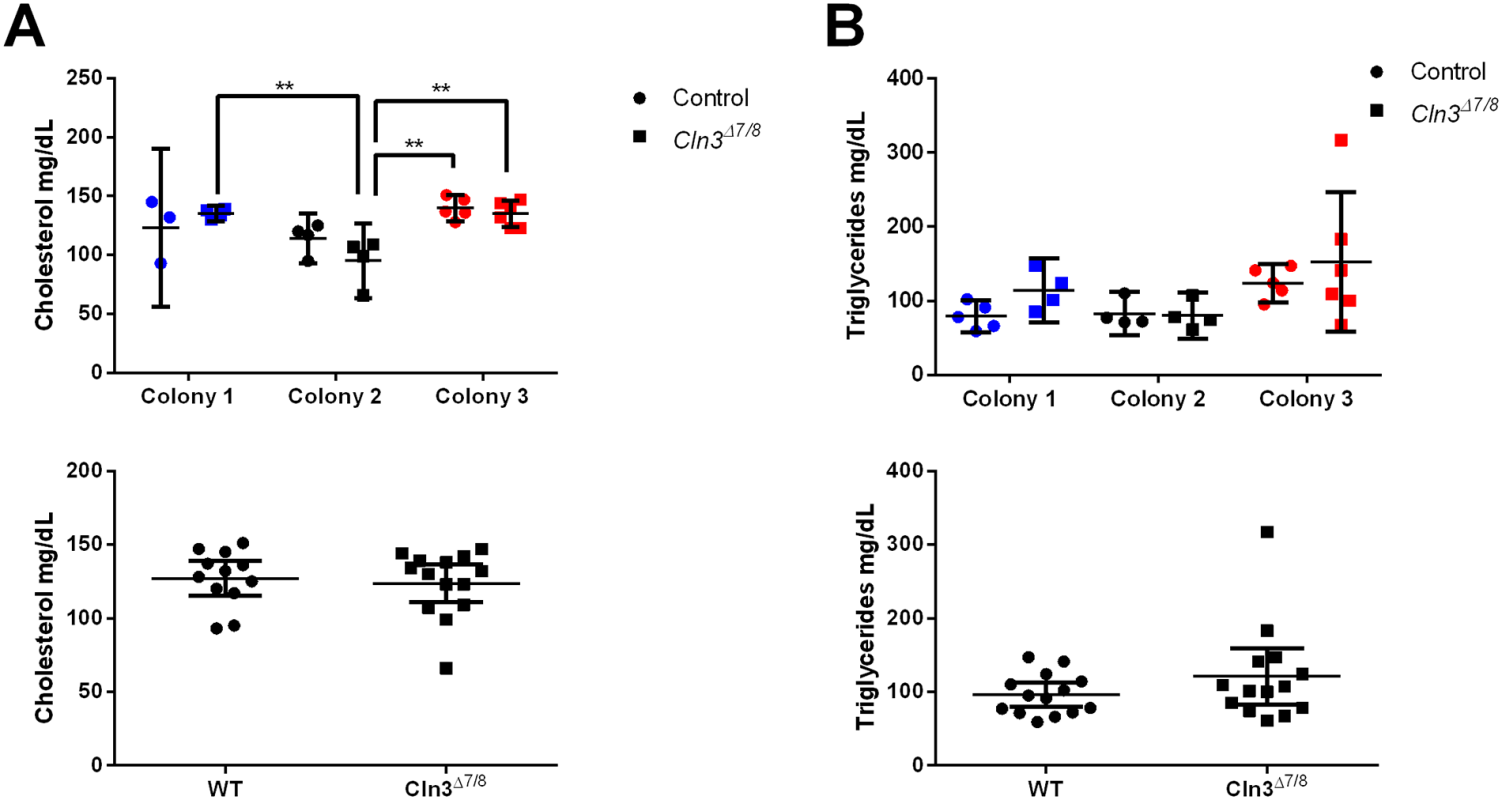
Colony specific effects on lipid levels in *Cln3*^*Δex7/8*^ and wild-type animals at 5 months of age. Cholesterol and triglyceride levels were analyzed for colonies 1 (blue, Sanford Research), 2 (black, University of Nebraska Medical Center), and 3 (red, Rosalind Franklin University of Medicine and Science) for both control (circle) and *Cln3*^*Δex7/8*^ mice (square). Graphs indicate concentration of cholesterol (A) and triglycerides (B), with individual colony data on top and pooled colony data on the bottom. There was a significant effect of the animal’s housing colony of measured cholesterol levels (p = 0.0003, A), and triglyceride levels (p-0.0344, B) Data represented as mean ± 95% CI; data points represent individual mice at indicated colony location. Statistical significance for top graphs was determined using ordinary two-way ANOVA, followed by Tukey’s multiple comparison test with a Bonferroni correction. Statistical significance for bottom, pooled colony graphs was determined using an unpaired student’s t test. **p<0.01.

Lipid metabolism of *Cln3*^*Δex7/8*^ mice was assessed by measuring blood levels of cholesterol and triglycerides. When the data was separated for individual colonies, there was a significant effect of the animal’s colony on cholesterol and triglyceride levels (p = 0.0003, p = 0.0344, respectively; Fig 2A and 2B). This is likely due, at least in part, to the difference in lipid concentration across different institution diets. When the data from all three colonies were pooled, neither cholesterol nor triglyceride levels were significantly altered in *Cln3*^*Δex7/8*^ mice (Fig 2A and 2B). Further study is needed to determine if *Cln3*^*Δex7/8*^ mice possess any lipid metabolism defects when presented with different diets or housing conditions.

### Comprehensive metabolic panel

**Fig 3:**
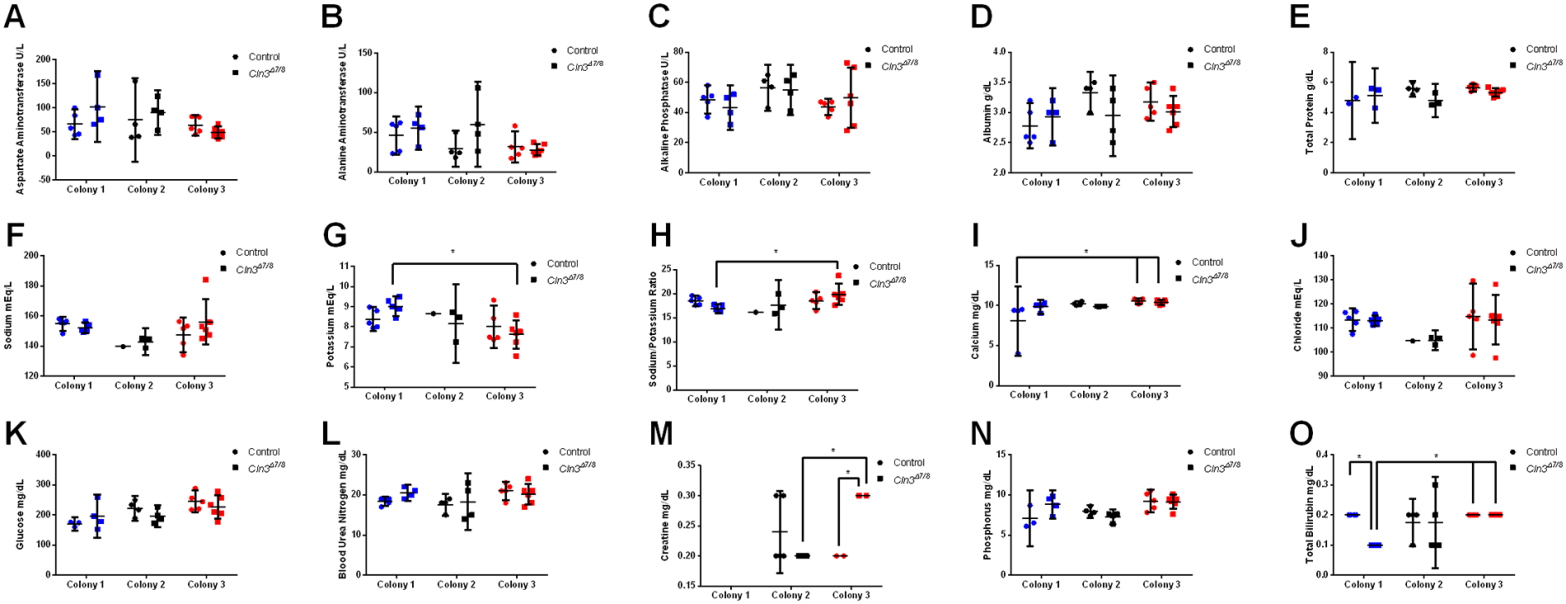
Colony specific effects and colony:genotype interactions in metabolic parameters between control and *Cln3*^*Δex7/8*^ mutant mice at 5 months of age. Metabolic panels measuring blood levels of aspartate aminotransferase (A), alanine aminotransferase (B), alkaline phosphatase (C), albumin (D), total protein (E), sodium (F), potassium (G), sodium:potassium ratio (H), calcium (I), chloride (J), glucose (K), blood urea nitrogen (L), creatinine (M), phosphorus (N), and total bilirubin (O) were analyzed for colonies 1 (blue, Sanford Research), 2 (black, University of Nebraska Medical Center), and 3 (red, Rosalind Franklin University of Medicine and Science) for both control (circle) and *Cln3*^*Δex7/8*^ mice (square). Colony specific effects were seen in glucose (p = 0.0090, K), total protein (p = 0.0439, E), calcium (p = 0.0184, I), phosphorus (p = 0.0045, N), potassium (p = 0.0186, G), and the sodium/potassium ratio (p = 0.0411, H), with an interaction between genotype and colony seen in total bilirubin (Int p = 0.0289, Colony p = 0.0493, Genotype p = 0.0484, O) and creatine levels (p = 0.0040, M). Data represented as mean ± 95% CI; data points represent individual mice from indicated colony. Statistical significance using ordinary two-way ANOVA, followed by Tukey’s multiple comparison test with a Bonferroni correction. *p<0.05.

Next, the metabolic state of *Cln3*^*Δex7/8*^ mice was evaluated by measuring the concentration of 15 different analytes: aspartate aminotransferase, alanine aminotransferase, alkaline phosphatase, albumin, total protein, sodium, potassium, sodium to potassium ratio, calcium, chloride, glucose, blood urea nitrogen, creatinine, phosphorus, and total bilirubin. Similar blood chemistry analysis has been performed on *Cln3*^*Δex7/8*^ mice as a readout of liver, kidney, and pancreatic function [24]. Analysis of the data as separate colonies (Fig 3A–3O) showed significant effects of the animal’s housing colony on glucose (p = 0.0090), total protein (p = 0.0439), calcium (p = 0.0184), phosphorus (p = 0.0045), potassium (p = 0.0186), and the sodium/potassium ratio (p = 0.0411). This is likely a result of the handling differences at each institution, such as diet composition. Interestingly, there was a significant interaction between colony and genotype on total bilirubin (p = 0.0289) and creatine levels (p = 0.0040)). When data from all three colonies was combined, we saw no significant differences in any of the measured parameters between 5 month-old *Cln3*^*Δ7ex7/8*^ and wild-type mice (**Panels A-O in** S1 Fig).

### Complete blood counts and hematological parameters

Blood collected from *Cln3*^*Δex7/8*^ and wild-type mice was analyzed for complete blood counts (CBCs) at 1, 3, 6, and 12 months-of-age in two colonies. When data was analyzed on a colony-by-colony basis, some significant variations were detected.

**Fig 4:**
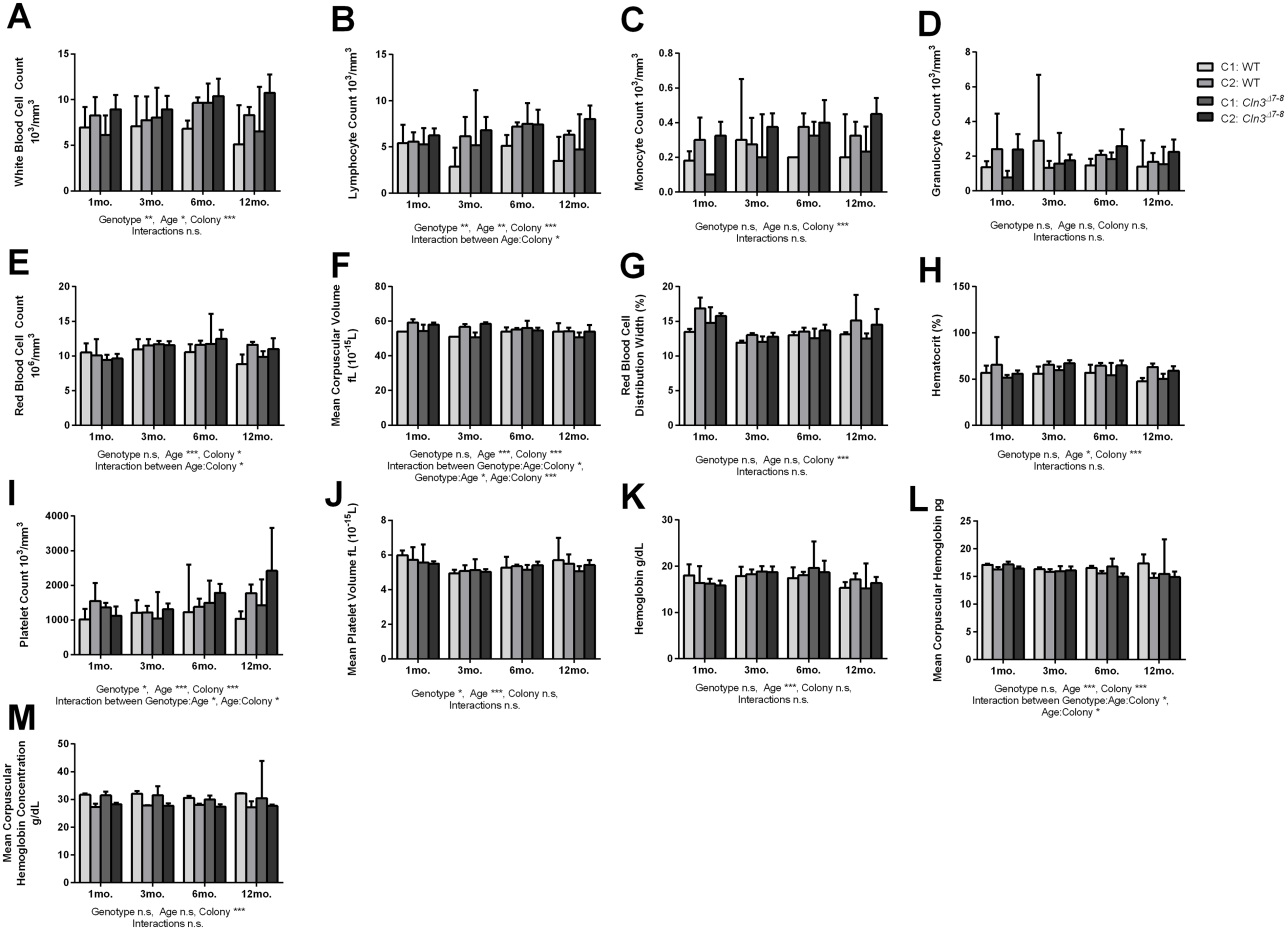
Effect of age, genotype, colony, and interaction on blood count parameters between separately housed wild-type and *Cln3*^*Δex7/8*^ mice. Blood collected from 1, 3, 6, and 12-month-old wild-type and *Cln3*^*Δex7/8*^ mice was assessed for differences in the number of total white blood cells (A), lymphocytes (B), monocytes (C), granulocytes (D), RBCs (E), mean corpuscular volume (F), red blood cell distribution width (G), hematocrit (H), platelet count (I), mean platelet volume (J), hemoglobin (K), mean corpuscular hemoglobin (L), and mean corpuscular hemoglobin concentration (M). Graphs are representative of mice from Sanford Research (colony 1) and The University of Nebraska Medical Center (colony 2). A significant effect of genotype across the two colonies was detected in white blood cell (p = 0.0011, A), lymphocyte (p = 0.0001, B), platelet (p = 0.0110, I), and mean platelet volume (p = 0.0215, J) metrics. There were a number of metrics where a significant effect of the animal’s housing colony or age was noted, which are detailed in S1 and S2 Files. There were a number of instances where a significant interaction between an animal’s housing colony, genotype, and/or age was detected, including red blood cell count (Age:Colony p = 0.0406, E), mean corpuscular volume (Genotype:Age:Colony p = 0.0216, Genotype:Age p = 0.0230, Age:Colony p = 3.5^−8^, F), mean corpuscular hemoglobin (Genotype:Age:Colony p = 0.0496, Age:Colony p = 0.0238, L), platelet count (Genotype:Age p = 0.0271, Age:Colony p = 0.0111, I), and lymphocyte count (Age:Colony p = 0.0120, B). Data represented as mean ± 95% CI; data points represent individual mice from indicated colony. Statistical significance was determined using ordinary three-way ANOVA, followed by Tukey’s multiple comparison test with p-values adjusted for multiple comparisons. *p<0.05, **p<0.01, ***p<0.001, ****p<0.0001.

**Fig 5:**
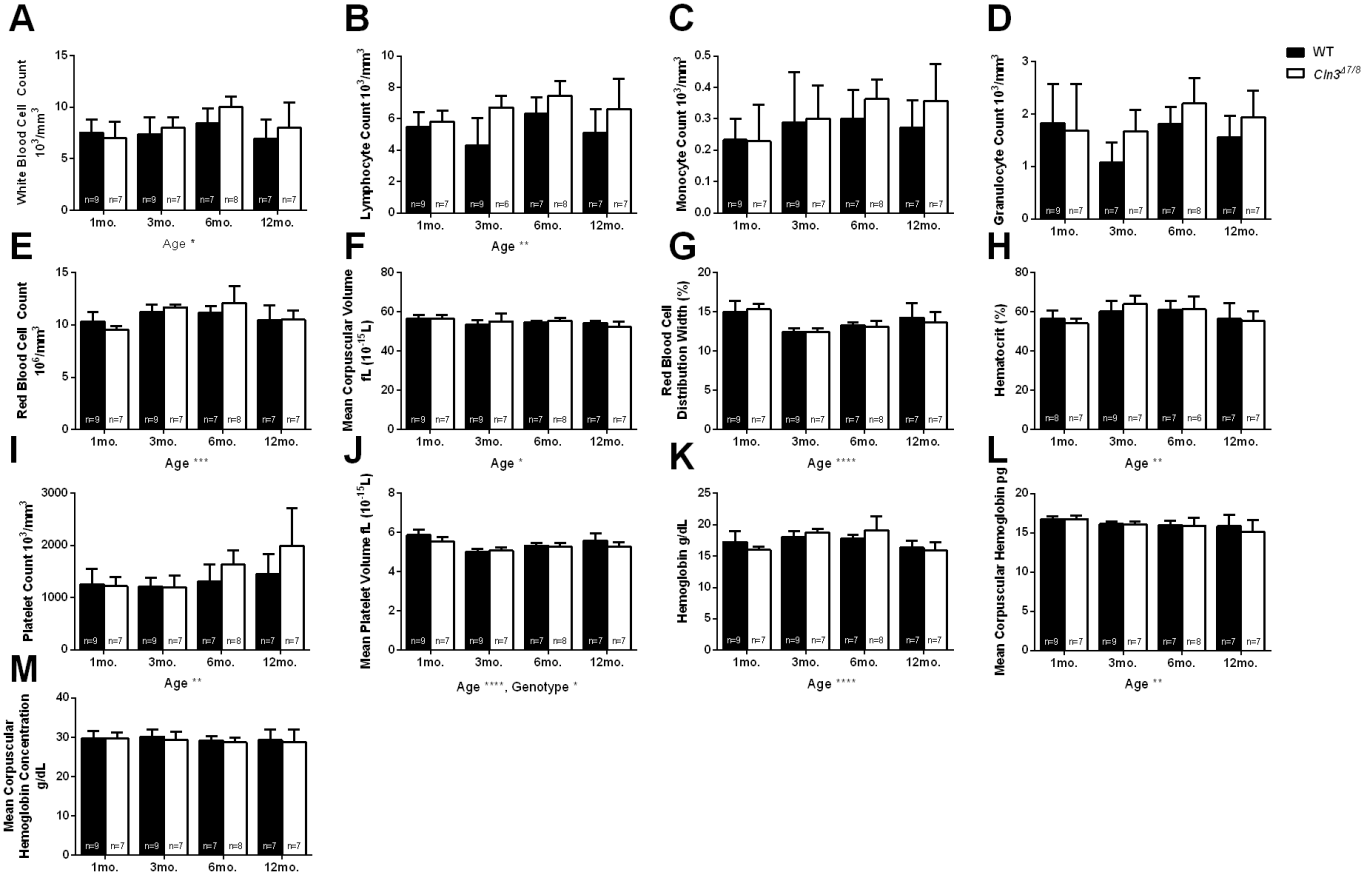
Effect of age, genotype, and interaction on blood count parameters between wild-type and *Cln3*^*Δex7/8*^ mice. Data from 1, 3, 6, and 12-month-old wild-type and *Cln3*^*Δex7/8*^ mice from Colony 1 and Colony 2 were pooled together and evaluated for differences in number of white blood cells (A), lymphocytes (B), monocytes (C), granulocytes (D), RBCs (E), mean corpuscular volume (F), red blood cell distribution width (G), hematocrit (H), platelet count (I), mean platelet volume (J), hemoglobin (K), mean corpuscular hemoglobin (L), and mean corpuscular hemoglobin concentration (M). A significant main effect of age was detected on several of the parameters measured and is detailed in S1 File. A significant main effect of genotype on mean platelet volume was also detected (p = 0.0019, J). Data represented as mean ± 95% CI; data points represent individual mice from indicated colony. Statistical significance was determined using ordinary two-way ANOVA, followed by Tukey’s multiple comparison test with a Bonferroni correction. *p<0.05, **p<0.01, ***p<0.001, ****p<0.0001.

A significant effect of genotype across the two colonies was detected in white blood cell (p = 0.0011), lymphocyte (p = 0.0001), platelet (p = 0.0110), and mean platelet volume (p = 0.0215). (Fig 4A, 4B, 4I and 4J). For the majority of the CBC metrics, there were a number of metrics where a significant effect of the animal’s housing colony or age was noted (Fig 4A–4L). Most importantly, there were a number of instances where a significant interaction between an animal’s housing colony, genotype, and/or age was detected, including red blood cell count (Age:Colony p = 0.0406), mean corpuscular volume (Genotype:Age:Colony p = 0.0216, Genotype:Age p = 0.0230, Age:Colony p = 3.5^−8^), mean corpuscular hemoglobin (Genotype:Age:Colony p = 0.0496, Age:Colony p = 0.0238), platelet count (Genotype:Age p = 0.0271, Age:Colony p = 0.0111), and lymphocyte count (Age:Colony p = 0.0120) (Fig 5B, 5E, 5F, 5I and 5J). These results indicate that CBC parameters may be inconsistent across time and colony when comparing wild-type and *Cln3*^*Δex7/8*^ mice. When data from the two colonies were pooled, there were no significant differences between aged matched wild-type and *Cln3*^*Δex7/8*^ mice in any of the parameters measured. There was, however, a significant main effect of age on several of the parameters measured, and a significant main effect of genotype on mean platelet volume (p = 0.0019) (Fig 5A, 5B, 5E–5L). These findings further support our conclusion that a consistent biomarker is difficult to find across multiple colonies of wild-type and *Cln3*^*Δex7/8*^ mice.

## Discussion

Compared to other lysosomal storage disorders, *CLN3*-Batten disease remains one of the more poorly understood in terms of underlying molecular mechanism; the exact function of CLN3 is unknown and with no known enzymatic function it is not amenable to enzyme replacement therapy or similar therapeutic approaches [25]. The *Cln3*^*Δex7/8*^ mouse model of *CLN3*-Batten disease has become an invaluable tool for studying disease pathology, as it recapitulates many of the phenotypic characteristics associated with human *CLN3*-Batten disease. However, identification of reproducible, non-invasive, biological surrogate marker(s) that will allow for the longitudinal monitoring of disease progression and readouts for therapeutic responses remain elusive. In this work, we utilized blood chemistry panels and complete blood counts to compare wild-type and *Cln3*^*Δex7/8*^ mice for differences that might be exploited as disease biomarkers. Importantly, we also determined how consistent these parameters were between distinct colony locations as the ideal biomarker would be reproducible notwithstanding subtle confounding factors imposed by study location.

There were a number of metrics that were influenced by colony location across both genotypes, though most importantly many of these were affected by an interaction of the genotype, colony, and/or age of the animals. These included ferritin (Fig 1C), total bilirubin and creatine (Fig 3M and 3O), red blood cell count, mean corpuscular volume, mean corpuscular hemoglobin, platelet count, and lymphocyte count (Fig 4B, 4E, 4F, 4I and 4L).

While a previous report by Staropoli et al has suggested altered serum ferritin levels in *CLN3*-Batten disease mice could be a useful biomarker, we did not find significant differences between our three colonies of wild-type and *Cln3*^*Δex7/8*^ mice[18]. Rather, we found a significant interaction of genotype and colony on ferritin levels (Fig 2C). It is worth noting that the absolute levels of ferritin, even in control mice, varied substantially between these two studies, though when combined with the interaction of genotype and colony revealed in our study, this suggests that ferritin may not be a consistent biomarker of *CLN3*-Batten disease. Similarly, while this previous study reported that MCV were elevated in *Cln3*^*Δex7/8*^ mice, no differences in MCV were detected between individual colonies of wild-type and *Cln3*^*Δex7/8*^ mice in our current study. Rather, a significant interaction between genotype, age, and/or colony was detected (Fig 4F). We note that the mice in the current study were older (5 months of age) at time of metabolic panel analysis than those tested in the earlier study from Staropoli et al. (12–19 weeks). This difference in the time of analysis may indicate that ferritin levels are elevated in *Cln3*^*Δex7/8*^ mice earlier in life and then stabilize as the mice age. It is also possible that strain differences play a role, as we used *Cln3*^*Δex7/8*^ mice in a C57BL/6J background, while Staropoli et al. used the C57BL/6N strain. Previous studies have documented phenotypic variation between the J and N inbred strains, including differences in clinical blood chemistry, hematology, metabolism, and immune function relevant to many of the parameters we analyzed here, as well as neurobehavioral differences [26]. Background selection has a significant impact on other disease markers, such as ocular lesions, confounding visual acuity tests commonly performed for Batten disease models [27]. Regardless of potential reasons for the discrepancy, in our study, there were no parameters we observed that showed both significant differences between mutant and wild-type mice and were observed consistently across multiple colonies.

Our results highlight the importance of standardizing the environment and likely the age of the animals as much as possible when searching for relevant biomarkers of genetic diseases. Geographical location of a colony and associated environmental factors have been shown to affect body weight, motor coordination, and learning capability in wild-type mice [19]. From the more obvious and easily controlled environmental factors such as diet, source of water, and bedding type, seemingly innocuous differences such as type of cage, enrichment, number of mice housed per cage, and differences in social hierarchies within cages are all environmental factors that can impact the results of animal studies [28–30]. Even changing position of cages on the rack can affect temperature, humidity, light and sound intensity a given cage is subject to [31]. Furthermore, animals being compared should come from the same source (commercial or otherwise) to minimize variability due to genetic drift. While these factors can be controlled to a great extent, different personnel handling mice at each location is one potential confounding variable that is difficult to control; in fact, in some behavioral studies, it has been demonstrated that even the gender of the human observer can affect baseline responses with respect to measures of pain and anxiety [32]. While the aforementioned studies refer mainly to how environmental factors can influence reproducibility in rodent behavioral experiments, importantly, other studies have reported that environmental and handling differences can significantly affect mouse physiological parameters such as blood chemistry. For example, number of mice housed per cage (2 vs. 4), timing of blood draw (morning vs. afternoon), venipuncture site (tail vein vs. retro-orbital), notably altered glucose, triglycerides, and/or iron are all potential variables [33, 34].

## Conclusions

In summary, we sought to elucidate reproducible, non-invasive, surrogate biomarkers in the *Cln3*^*Δex7/8*^ mouse model of *CLN3*-Batten disease that could potentially be translated to human patients. Of all the blood chemistry and hematological read outs we measured, there were no parameters that were consistently altered between wild-type and *Cln3*^*Δex7/8*^ mice across multiple colonies. Further, our findings were inconsistent with previously reported changes reported between wild-type and *Cln3*^*Δex7/8*^ mice [18]. A major unmet need for CLN3-Batten research, epidemiology, and therapeutic development is a robust, biologically relevant biomarker that can be tracked longitudinally in human patients, where environmental variations are much more substantial and far less controllable than in mice. Our results suggest that there is not a robust biomarker for CLN3-Batten disease in commonly tested hematological measurements.

## Supporting information

S1 Fig No changes in metabolic parameters between control and *Cln3*^*Δex7/8*^ mutant mice at 5 months of age. Metabolic panels measuring blood levels of aspartate aminotransferase (A), alanine aminotransferase (B), alkaline phosphatase (C), albumin (D), total protein (E), sodium (F), potassium (G), sodium/potassium ratio (H), calcium (I), chloride (J), glucose (K), blood urea nitrogen (L), creatinine (M), phosphorus (N), and total bilirubin (O) were pooled for all wild-type and *Cln3*^*Δex7/8*^ mice. No differences were observed in any of the molecules when comparing wild-type to *Cln3*^*Δex7/8*^ mice. Data represented as mean ± 95% CI; data points represent individual mice (pooled from all three colony locations). Statistical significance was determined using an unpaired, two-tailed, Student’s t-test.
(TIF)

S1 File Statistical results table from two-way ANOVAs—CBCs. (XLSX)

S2 File Statistical results table from three-way ANOVAs—CBCs. (XLSX)

S3 File Raw data. (XLSX)
